# The Combination of Antagonistic Drugs

**Published:** 1897-12-25

**Authors:** 


### THE COMBINATION OF ANTAGONISTIC DRUGS.

However great may be the compensatory changes by aid of which the circulatory difficulties occurring in the earlier stages of arteriosclerosis are overcome, a time sooner or later arrives when they break down by failure of the heart. When this happens, the question of treatment becomes a very urgent one. Mere vaso-dilators, useful as they are in giving relief to symptoms of cardiac distress, too often, it is to be found, produce their beneficial effects at the expense of lowering the blood pressure by virtue of which the tissues are fed; while, on the other hand, digitalis, the drug upon which our greatest reliance is placed for the purpose of bracing up the heart, at the same time contracts the blood-vessels, and in this way actually increases the difficulty that has to be overcome.

Dr. C. R. Marshall, of Cambridge, in a paper recently published, points out that in the course of this disease the centre of interest changes, so that what was a "vascular becomes a heart affection."

From a therapeutic point of view, he says, the condition may be divided into three stages:—

1. Early stage—thickened arteries, with slight enlargement (hypertrophy) of the heart; increased intra-arterial blood pressure.
2. Later stage—arteries somewhat thicker; marked cardiac hypertrophy; high arterial blood pressure.
3. Last stage—arteries unchanged or somewhat dilated, but heart failing; marked cardiac dilatation; gradual sinking blood pressure.

It is probable enough that if one could go to the origin of things it might be discovered that this gradual thickening of the whole arterial tree was the outcome of some chemical change in the circulating fluid, a toxæmia, perhaps resulting from deficient elimination, perhaps from error of diet or abnormalities of digestion and metabolism; and these things, if discovered in time, might perhaps be capable of rectification. But, as a fact, by the time the patient comes under treatment the arteries have always become changed, and in too many instances the heart has already begun to fail. According as the one (the first) or both of these conditions are present, so will the treatment have to be directed—in the one case to preventing the occurrence of those periods of high tension which, by their repetition, are among the more active causes of the early occurrence of heart failure; and, in the other, to counteracting the effects of the failure itself.

Here, then, we come to the question whether digitalis, a drug so useful in intrinsic failure arising in the heart itself, should be given in failure arising from what we may call over-load—from its constantly working against a thickened and obstructive arterial system—in view of the fact that digitalis itself contracts the arteries, and thus increases the obstruction? Another question is almost equally important, viz., To what extent are we justified in making use of vaso-dilators for the relief of tension when on the maintenance of that tension the feeding of the tissues depends?

There is plenty of evidence that the mere administration of digitalis is often useless in the presence of high tension, and, on the other hand, that vaso-dilators can only be used with safety for purely temporary purposes when the circulation is already failing from cardiac insufficiency. Many physicians, however, have adopted the plan of giving vaso-dilators along with digitalis, notwithstanding the apparent antagonism of their action; giving them not with the object of lessening the tension that exists, but of preventing that excess of tension which might be caused by the action of the digitalis itself upon the arteries.

In regard to this, Dr. Marshall says that just as the only, or almost the only indication for the use of digitalis is a failing heart, so the only indication for the use of vaso-dilators is a more or less incompressible pulse. Wherever the two are combined—that is, wherever a comparatively high tension pulse exists with heart failure, the combination will be of use. But he gives a warning in regard to the mode of administration; for while digitalis is a slow-acting remedy, the vaso-dilators act quickly, and therefore they should not be given together. When digitalis has once got to work one dose a day is often sufficient to keep up its operation, but, "to keep down the tension of the pulse nitroglycerine and sodium nitrate should be given at least every three hours, and erythrol tetranitrate every six. Given less frequently the patient is periodically subjected to periods of high arterial tension of varying duration, which is what we wish to avoid. . . . If I might suggest a type of combination, it would be the administration of erythrol tetranitrate every six hours, and one of the French granules of digitalin every one, two, or three days, according to the progress of the disease and the effects of previous treatment." Dealing with the same subject, but from a different standpoint, Dr. Arthur Wilkinson, in his presidential address to the Manchester Clinical Society, says that, although the action of certain drugs may be so antagonistic that they may be considered antidotes to one another, still they may not be co-extensive in action, and they may in therapeutics act as "correctives." Thus, although in cardiac failure the action of digitalis is far more useful than that of any other drug, its liability "to raise the arterial pressure by increasing the peripheral resistance makes its unqualified use hazardous when the arterioles in places are unfit to bear the strain. We therefore require something to mitigate this action of the drug whilst upholding or increasing its diuretic power. In my experience," he adds, "iodide of potassium possesses this property, and digitalis may be safely administered in combination with the iodide when it might be dangerous to give it alone."

No doubt there are other drugs by which somewhat the same effect may be obtained. In many pharmaceutical compounds substances are added as correctives, substances which are antagonistic to some of the properties of the main ingredient, while not interfering with the special action for the sake of which it is given, and when a vaso-dilator is ordered along with digitalis we have but another example of the same method, even though at first sight the actions of the two drugs may seem entirely opposed to one another.

